# First case of intestinal acariasis from Egypt

**DOI:** 10.1186/s40064-015-1584-4

**Published:** 2016-01-12

**Authors:** Refaat M. A. Khalifa, Manal Z. M. Abdellatif, Azza K. Ahmed, Doaa A. Yones, Abdel-Azeem M. El-Mazary, Lamia H. Aly, Mahmoud A. El-Seify, Moustafa A. Haridi

**Affiliations:** Medical Parasitology Departments, Faculty of Medicine, Assiut University, Assiut, Egypt; Minia University, Minia, Egypt; Pediatric Department, Faculty of Medicine, Minia University, Minia, Egypt; Clinical Pathology Department, Faculty of Medicine, Minia University, Minia, Egypt; Parasitology Department, Faculty of Veterinary Medicine, Kafr Elsheikh University, Kafr El-Sheikh, Egypt; Internal Medicine, Faculty of Medicine, Assiut University, Assiut, Egypt

**Keywords:** Intestinal infection, *Tyrophagus putrescentiae*, Mite, *Blastocystis* sp., Young boy

## Abstract

We are hereby reporting a case where the eggs and adults of the mold mites; *Tyrophagus putrescentiae* (Shrank) and the trophozoites of *Blastocystis* sp. were found in stool of three years old child from Minia City, Egypt. Intestinal mite infection was diagnosed after repeated identification of mite’ stages from six consecutive stool samples to exclude the possibilities of contamination and spurious infection. The patient was suffering from severe colicky abdominal pain and burning sensation around the anus one month ago. All other members of his family were having the same acarine in their feces, but were all symptomless. The patient was treated with ivermectin 200 µg/kg body weight once every 10 days for three doses. His cure indicated that he was having asymptomatic blastocystosis.

## Background

House dust mites represent a large group of subclass Acari, belonging to the suborder Acaridida of the order Acariformes. They can be detected in dust and vacuum samples from floors, furniture, mattresses, dry fruit, grain, flour, sugar, and bedding. These mites feed on sloughed human skin, fungi, spilled food and pollen (Li and Wang, 2000). Domestic mite species are usually found inside houses in warm and tropical regions. They are known for causing allergic disorders. However, little information is available about human acariasis, in which mites invade different tissues of human body as gastrointestinal, urinary and respiratory tracts (Cui 2014).

## Case study

A three years old Egyptian male child, from Minia City, presented to the outpatient pediatric clinic, Minia University Teaching Hospital. His parents stated that the child was complaining of severe colicky abdominal pain, and burning sensation around the anus one month ago. The pain was all over the abdomen and not related to meals. His parents observed perianal redness accompanied by loss of weight and anorexia. There was no vomiting, hematemesis, diarrhea or constipation. Physical examination revealed good general condition, alert and cooperative child. There was no jaundice, pallor or cyanosis. The vital signs as well as the anthropometric measurements were normal. There was no lymph node enlargement or edema of both lower limbs. The abdominal examination revealed generalized abdominal tenderness without organomegaly or ascites. Blood examination revealed normal complete blood count except mild normochromic normocytic anemia (HB % 9.4 gm/dL), slightly elevated liver enzymes (AST 78 and ALT 98 IU/L), normal serum amylase (47 IU/L), lipase (17 IU/L) and alkaline phosphatase (220 IU/L) enzymes. The kidney functions tests (urea 24 mg/dL and creatinine 0.7 mg/dL) and urine examination were normal. Stool samples were referred from a routine laboratory to the Parasitology Department, Faculty of Medicine, Minia University for investigation. Also the patient was referred to Medical Parasitology Department, Faculty of Medicine, Assiut University, for stool examination and mite identification.

The child data were taken and used in the study after an informed signed consent was taken from his father.

### Macroscopic examination

The stool was grayish in color, offensive odor, no mucous or blood and semi-formed in consistency. Stool was processed for routine microscopy by preparing saline and iodine preparations.

### Microscopical examination

**Fig. 1:**
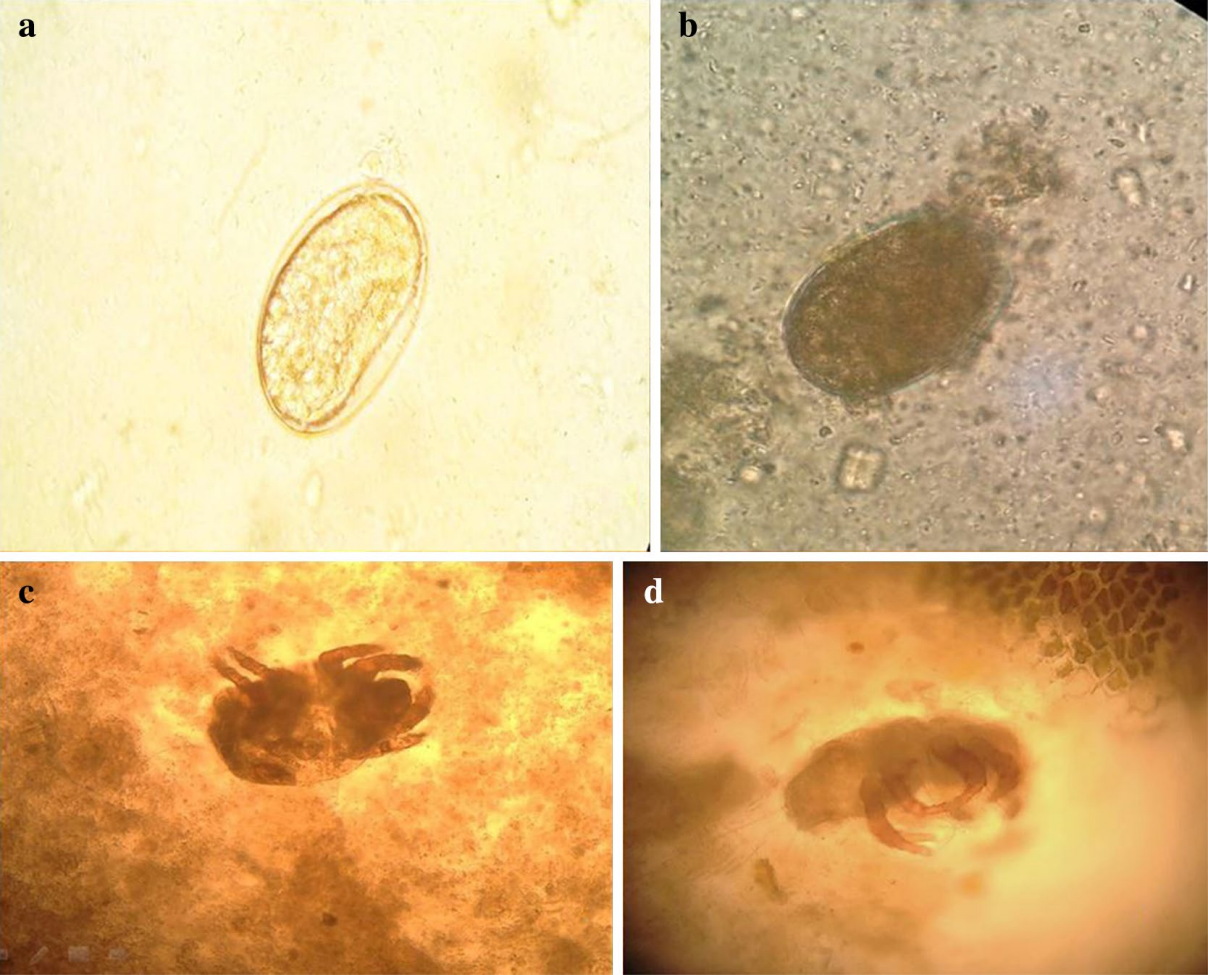
**a** Photomicrograph of oval egg with smooth outline (×10). **b** Photomicrograph of oval eggs with heavily ornamented surface (×10). **c** Adult eight-legged mite is partially transparent (×10). **d** adult mite with four long sub-equal hairs on the abdomeninal end (×20)

The microscopical examination showed multiple large-sized oval eggs measuring 105–110 by 72–75 µm. Some eggs were having smooth outlines while others were having rough shells and heavily ornamented surface. The egg was measuring 110 μm (Fig. 1 a, b). Eight-legged adult mites measuring 300–400 µm were also found (Fig. 1 c, d). For mite’s identification, they were isolated by flotation on saturated saline and identified microscopically (Malainual et al. 1995). Mites’ body was semi-transparent with subequal long four train hairs on the abdomeninal end. The mites were identified according to Gamal Eldin et al. (1982) and Gamal Eldin and Hamad (1992) to be identical with *Tyrophagus putrescentiae*. It is worth mentioning that stool examination of all the family members proved positive for the same mite adults and eggs, but surprisingly all of them were not complaining from any symptoms.

Microscopic examination of the patient’s stool samples revealed also the presence of *Blastocystis* sp. trophozoites; mainly vacuolated forms with >5 parasites per HPF (high power field). These forms were measuring 4–15 µm and appearing as spherical or ovoidal cells with large single vacuole and multiple nuclei (up to 4) could be seen in the surrounding cytoplasm.

The similarity of shape and size between the mite undeveloped eggs and helminth eggs led to false diagnosis of hookworm eggs by the routine laboratory technicians and thereby erroneous treatment by Mebendazol syrup (Bendax) one teaspoonful twice daily for 3 days failed. Hence, the stool samples were referred to the laboratory of the Medical Parasitology Departments for more accurate identification; where the mites and their eggs were recognized and thereafter, acariasis was treated by ivermectin (Iverzine 6 mg-UNIPHARMA-Cairo, Egypt). He received single dose; 200 µg/kg body weight and one teaspoonful of castor oil per day and repeated every 10 days for three consecutive doses after which stool examination revealed no eggs and fragmented parts of adults. The patient’ symptoms subsided, started to gain weight and his stools were parasite free. Follow up of patient’s symptoms and stool examination revealed no recurrence.

## Discussion

In this study the identified mite in the patient’s faeces was belonged to *T. putrescentiae*. as both sexes were pear-shaped having four long hairs at the back end. The eggs were smooth when they start to develop then become heavily ornamented with bands of tubular microsculpture that protrude from surface of the shell (Huges 1976; Colloff and Spieksma 1992). Contamination of stool and spurious infection were excluded by repeated stool sampling, using clean stool containers.

*Tyrophagus* acaridae are generally fungivorus commonly found in stored food and decaying organic matter (Fan and Zhang 2007). *T. putrescentiae* was commonly identified in Egyptian houses; particularly in the kitchen (Gamal Eldin et al. 1989; Gamal Eldin and Shkair 1990; Gamal Eldin and El-Basheer 1991; Mowafy and Khalifa 1997; Khalifa et al. 2000).

Intestinal acariasis is rather an uncommon human disease; the first case was reported by Hinman and Kampmeier (1934) which was caused by *Tyroglyphus longior* Gervals. Kampmeier and Hinman (1934) had reported another two patients with diarrhea due to “intestinal acariasias” due to the same mite. Xing (1990) reported three cases of intestinal acariasis in China due to *T. putrescentiae*. Li and Wang (2000) studied cases of intestinal acariasis in Anhui Province, China from 1989–1996 and out of 3416 fecal sample examined, reported 94 with only mite infections and 131 cases together with other intestinal parasites. Li et al. (2003) reported 92 out of 1994 (4.61 %) stool samples positive for intestinal acariasis in China. Among others, *T. putrescentiae* caused the disease. Exceptionally an allergic intestinal acariasis syndrome has been described by Scala (1995). Zia et al. (2014) reported a case of intestinal acariasis in a 56 years old gentleman who was completely asymptomatic, but they did not identify the causative mite. Revising all the available literature from Egypt, we could not come across any encountered previous case.

As what happened in the present study, mite eggs in stool samples, were sometimes erroneously identified as helminth eggs, leading to false treatment with extensive consequences (Werneck et al. 2007; Zia et al. 2014). In the present study, mite’s eggs were at first misdiagnosed as hookworm eggs which were followed by unsuccessful antihelminthic treatment. Realizing the identity of the causative mite was followed by successful treatment with ivermectin as this broad spectrum antiparasitic drug was suggested by Li (2000) to be the drug of choice for human intestinal acariasis. It is worth mentioning that in our study, no treatment for blastocystosis was given and the cure of the patient denoted that the infecting *Blastocystis* sp. was non-pathogenic.

The asymptomatic infestation of the entire child’s family may be explained by the very young age of the patient. Furthermore, Zia et al. (2014) reported that their 56 years old mite carrier was quite healthy and concluded that no rigorous antiparasitic therapy was necessary to eliminate the dust mites from his system.

As the present mold mite is a common pest of stored food products and because temperature is an essential factor that limits the survival of the arthropod species; Eaton and Kells (2011) proved that 90 % of all mite stages of *T. putrescentiae* can be controlled within commodity or packaged products by freezing to −18 °C for 5h.

In the present study, intestinal acariasis was accompanied by infection with *Blastocystis* sp. which was found to be non-pathogenic. Members of the genus *Blastocystis* comprise several subtypes (genotypes) and it is suggested that its pathogenicity is related to specific subtypes and parasite burden (Sheehan et al. 1986; Stensvold et al. 2007; Al-kaissi and Al Magdi 2009; Coyle et al. 2011). There has been debate in the literature concerning the question of this organism pathogenicity; some studies suggest an association between the parasite and disease but others do not (Tan 2008). Most recent studies focused on correlating disease pathogenicity with subtypes irrespective of parasite density (Stensvold et al. 2009; Yakoob et al. 2010).

## Conclusion

Acaroid mites in our cases may invade human intestines and cause variable manifestations starting of being symptomless to anaemia and weight loss.

It is suggested that separation of mites from stool samples, skin prick test and detection of total IgE and mite-specific IgE should be used in the diagnosis of acaroid mites. Intestinal acariasis may be easily misdiagnosed for allergic enteritis, chronic colitis, intestinal neurosis, amoebiasis, pelvic inflammation, or schistosomiasis. As a result, proper treatment might be thus delayed. Clinicians and technicians should always bear in mind the possibility of dust mite infestation while examining suspicious specimens. This is important in regions, like ours, where intestinal parasites are present so as to avoid unnecessary and ineffective treatment.

## Patient consent

The patient’s father has consented to the submission of the case report for submission to the journal.
